# Kininogen enhances seizure susceptibility in mice possibly through bradykinin-induced modulation of calcium transients in glutamatergic and GABAergic neurons

**DOI:** 10.3389/fphar.2025.1509837

**Published:** 2025-06-10

**Authors:** Arijit Ghosh, Jiwen Huang, Qian Wang, Yanling Gong, Srishti Jain, Abhimanyu Thakur, Amiyangshu De, Meixi Gong, Yoshie Kiyohara, Jingtai Hu, Lingyan Jin, Sharba Bandyopadhyay, Fang Chen, Shengtian Li

**Affiliations:** ^1^ Bio-X Institutes, Key Laboratory for the Genetics of Development & Neuropsychiatric Disorders (Ministry of Education), Shanghai Jiao Tong University, Shanghai, China; ^2^ School of Life Sciences and Biotechnology, Shanghai Jiao Tong University, Shanghai, China; ^3^ Advanced Technology Development Centre, Indian Institute of Technology Kharagpur, West Bengal, India; ^4^ Information Processing Laboratory, Department of Electronics and Electrical Communication Engineering, Indian Institute of Technology Kharagpur, Kharagpur, West Bengal, India; ^5^ Department of Pharmacology, Delhi Pharmaceutical Sciences and Research University, Delhi, India; ^6^ Department of Neurosurgery, Massachusetts General Hospital, Harvard Medical School, Boston, MA, United States; ^7^ No. 2 High School of East China Normal University, Zizhu, Shanghai, China; ^8^ Shanghai Fifth People's Hospital, Fudan University, Shanghai, China; ^9^ Department of Pharmacy, the First Affiliated Hospital of Xiamen University, Xiamen, China

**Keywords:** calcium imaging, parvalbumin, pentylenetetrazole, pilocarpine, PV^Cre^-GCaMP6f, seizure imaging, Thy1-GCaMP6f

## Abstract

**Background:**

Previously we showed that elevated cerebrospinal fluid (CSF) levels of kininogen during the acute phase of encephalitis are associated with symptomatic epilepsy. However, the functional role of kininogen in epileptogenesis remains unexplored.

**Objective:**

This study investigated the brain expression of kininogen and its influence on seizure susceptibility. Additionally, we examined the effects of bradykinin, a nonapeptide derived from kininogen, as a potential downstream mediator of kininogen's effect on seizure susceptibility and the underlying circuitry mechanisms.

**Methods:**

We analyzed brain mRNA expression of kininogen using publicly available single-cell RNA-sequencing (scRNA-seq) datasets and assessed protein expression through immunofluorescence in various brain regions. Immunoblot was conducted following pilocarpine-induced status epilepticus (Pilo-SE) to understand post-seizure kininogen dynamics. Next, to understand its functional role, kininogen was overexpressed in the hippocampal CA1 area via its AAV-mediated gene delivery, and bradykinin was administered through the fourth ventricle in mice. The effects on seizure susceptibility were evaluated using a pentylenetetrazole-induced seizure susceptibility test. Furthermore, two-photon *in vivo* calcium imaging of cortical layer 2/3 glutamatergic neurons and GABAergic parvalbumin-positive neurons was performed to explore the potential circuitry mechanisms.

**Results:**

While scRNA-seq analyses found kininogen gene expressions in various cell types across the brain, immunofluorescence revealed its preferential localization in neurons but not in glia. Pilo-SE decreased intact kininogen levels in the hippocampus and increased cleaved to intact kininogen (cHK / iHK) ratio. Overexpression of kininogen and exogenous bradykinin administration significantly increased pentylenetetrazole-induced seizure susceptibility in mice. Mechanistically, bradykinin was found to enhance calcium activities in cortical glutamatergic excitatory neurons in Thy1-GCaMP mice when they were treated with a subthreshold dose of pentylenetetrazole. In contrast, calcium activities in GABAergic parvalbumin-positive inhibitory neurons were reduced by bradykinin in PV^Cre^-GCaMP mice, suggesting potential circuitry mechanisms by which kininogen may render increased seizure susceptibility.

**Conclusion:**

Our findings reveal a novel pathological role of kininogen in seizure occurrence, explaining why kininogen might be elevated in the CSF of epilepsy-susceptible patients and suggest its potential mechanisms where it might regulate the activities of glutamatergic and GABAergic neurons through the downstream release of bradykinin. Altogether, we propose kininogen as a potential target for developing therapeutics for epilepsy intervention.

## 1 Introduction

Epilepsy is one of the most devastating neurological diseases, affecting tens of millions of patients worldwide. Despite the availability of several antiepileptic drugs (AEDs), drug resistance in a quarter of the patients (Zou et al., 2019) poses significant challenges. Moreover, some AEDs can suppress only symptomatic seizures, while the preventive effects of these drugs against epileptogenesis are uncertain. Phenytoin and carbamazepine, for example, can reduce the incidence of early seizures but not chronic seizures following traumatic brain injuries (TBI) (Thompson et al., 2015; Fordington and Manford, 2020). Seizures occur in a significant proportion of TBI patients, with estimates suggesting that between 10% and 50% of these individuals will experience seizures at some point in their lifetime (Englander et al., 2014). The delay between the first injury and the onset of epilepsy is known as the period of epileptogenesis, which presents an opportunity to understand the underlying changes in the brain and explore potential therapeutic targets to counter the development of epilepsy (Fordington and Manford, 2020). Several detrimental processes, including inflammation in the early phases of TBI and some other factors in the later phases, contribute to circuitry changes that subsequently result in epilepsy (Fordington and Manford, 2020; Pitkänen et al., 2019). Another major contributor to epilepsy is encephalitis, which may cause seizures in as high as ≥ 67% of patients suffering from it (Michael and Solomon, 2012). Inflammatory responses following such infections are the main driving force for epileptogenesis (Spatola and Dalmau, 2017), which may be of either local brain origin or of peripheral origin (Mukhtar, 2020). A growing body of evidence suggests that proinflammatory cytokines can activate both the innate immune system and the adaptive immune system in epilepsy (Vezzani et al., 2016; Zhang, 2020), and a cascade of inflammatory processes involving all the major brain cell types cause imbalance in excitatory and inhibitory neurotransmission (Spatola and Dalmau, 2017). Moreover, inflammatory peptides produced by the brain-resident cells may directly promote excitatory neurotransmitter release and subsequent neuronal depolarization (Michael and Solomon, 2012). Therefore, targeting pathways and related inflammatory proteins/peptides early in the post-injury or infectious stage may pave the way for preventing epileptogenesis.

Kininogen is one of the major biological molecules that in humans regulates many pathophysiologic conditions, including inflammation (Köhler et al., 2020). It is a part of the kallikrein-kinin system (KKS), where it produces the inflammatory peptide bradykinin and other related kinins by the action of kallikreins. In the human, it is encoded by the *KNG1* gene while in mice it is encoded by two genes: *Kng1* and *Kng2*. While *mKng2* does not produce enough kininogen (Yang et al., 2017), the *mKng1* gene through alternative splicing translates to two major subtypes: a 110–120-kDa high-molecular-weight kininogen (HK), which is also known as the intact HK (iHK), and a 65–68-kDa low-molecular-weight kininogen (LK). HK is cleaved by the plasma kallikrein in the plasma, while LK is cleaved by another kallikrein family in the tissues. Cleavage of HK causes the liberation of bradykinin, while the cleavage of LK releases kallidin, which consists of an extra lysine residue compared to bradykinin and thus is also known as lysyl-bradykinin (Igić, 2018). Interestingly, kallidin is convertible to bradykinin through the actions of aminopeptidases (Igić, 2018). In the human plasma, HK has been reported to be present in a range of 65–130 μg/mL, while that of LK it has been reported to be in a range of 164–183 μg/mL (Hoem et al., 1989). Despite the fact that LK is known as the tissue kininogen, its concentration in the plasma of healthy subjects is much higher than that of the HK, as revealed by multiple studies (Hoem et al., 1989; Adam et al., 1985). In diseased conditions too, both plasma and CSF levels of LK can be higher than their respective HK levels (Dellalibera-Joviliano et al., 2003). However, plasma levels of HK or LK may not vary among males and females (Uchida, 1983). Except for the kallikreins, kininogens are also cleaved by other enzymes such as plasmin which mediate their cleavage activity through different cleavage sites other than the one used by the kallikreins (Dickeson et al., 2024). Of note, HK has several degradation products in the range of ∼38 kD to ∼65 kD that are left after the cleavage of the iHK, reported by several studies (Henderson et al., 2021; Kerbiriou and Griffin, 1979; Reddigari and Kaplan, 1988; Motta et al., 1998; Simões et al., 2019; Scott et al., 1984; Yamamoto-Imoto et al., 2018). A heavy-chain product of HK (HK-HC) is found at ∼62–65 kD (Reddigari and Kaplan, 1988; Merkulov et al., 2008) that shares a near-similar molecular weight with that of LK (Köhler et al., 2020; Schwartz et al., 1986). Then is found the light-chain product of HK (HK-LC) at ∼56 kD (Merkulov et al., 2008). A light-chain product of LK is also known to exist at about 4 kD (Merkulov et al., 2008). Two more degradation products of HK are found at ∼45–49 kD and 38 kD (Köhler et al., 2020; Scott et al., 1984; Yamamoto-Imoto et al., 2018). All these four degradation products of HK are known as the cleaved HK (cHK) (Henderson et al., 2021). Another cHK product in human plasma under certain diseased conditions have also been shown at a position of ∼100 kD (Suffritti et al., 2014). Interestingly, the degradation product of ∼62–65 kD is convertible to the 45-49 kD subtype by the action of plasma kallikrein while tissue kallikrein has no effect on such conversions (Reddigari and Kaplan, 1988). Another subtype of 82-kDa HK (ΔmHK-D5) which lacks the domain 5 (D5) and some proximal parts of the D6 is also known to exist (Merkulov et al., 2008). Kininogen is a part of the cystatin family and is traditionally known to inhibit cysteine/thiol proteases (Adenaeuer et al., 2023). Functionally, HK is mainly known to participate in the blood coagulation cascade (Ponczek, 2021), influence leukocyte and endothelial cell adhesion (Adenaeuer et al., 2023), and liberate bradykinin. Adhesion of HK on endothelial cells are facilitated by several types of proteoglycans (Renné and Müller-Esterl, 2001; Renné et al., 2000; Renné et al., 2005) - a phenomenon that may be important for factors such as angiogenesis, apoptosis, and fibrinolysis. Although the pathological effect of kininogen is majorly studied in peripheral diseases and infections (Adenaeuer et al., 2023), some data also highlights its association with and role in brain-related diseases. Some association of kininogen, for example, was found in Alzheimer’s disease (Yamamoto-Imoto et al., 2018), Parkinson’s disease (Markaki et al., 2020) and schizophrenia (Yang et al., 2019) patient brains. Deletion of *mKng1* was found to render protection from thrombosis, blood-brain barrier (BBB) damage, and inflammation, thus causing a reduction in ischemia-induced neurodegeneration in mice (Langhauser et al., 2012). In some brain diseases, bradykinin has also been shown to mediate BBB disruption (Gauberti et al., 2018; Marcos-Contreras et al., 2016) and modulate other factors (Gauberti et al., 2018), thus further aggravating the disease conditions. Bradykinin, which is a very short-lived peptide, mediates its effects mainly through two G-protein-coupled receptors (GPCR), namely, bradykinin receptor 1 (B1R) and bradykinin receptor 2 (B2R). While most studies involving brain diseases highlighted the role of these two receptors, a very few studies have reported on the direct role of kininogen or bradykinin in brain pathologies, warranting for more attention toward this domain.

One of our previous studies found that during the early epileptogenesis period in the lithium chloride-pilocarpine (LiCl-Pilo) model of rats, the cerebrospinal fluid (CSF) levels of kininogen are increased nearly by 2.0-fold while also increased in the hippocampus, an important brain area related to the pathogenesis and spread of seizures, by 4.0-fold compared to the Naïve littermate controls (Zou et al., 2019). Validating the same in encephalitis patients, it was found that a chunk of these patients who eventually developed symptomatic epilepsy within years from the onset of infection had their CSF kininogen levels elevated in the acute phase of the infection by ∼1.5-fold compared to the ones who did not develop chronic seizures (Zou et al., 2019). A proteomic analysis from another research group also found increased kininogen levels in the hippocampus of LiCl-Pilo-treated rats (Wang et al., 2022). Similarly, another study found that the activity of plasma kallikrein is elevated in the sera of epilepsy patients, contributing to cleavage of HK and thus upregulating both circulating and hippocampal levels of bradykinin (Simões et al., 2019). However, all these studies only suggest elevated levels and activity of kininogen or bradykinin in the circulation or brain tissues, while the functional role of these components in the context of seizures or epilepsy has not been yet explored. Moreover, functional studies related to the effects of B1R/B2R in experimental epilepsy have shown conflicting outcomes. Knockout of B2R, for example, in one study was shown to reduce latency and increase the frequency of spontaneous seizures, while the opposite effects were seen in animals with a knockout of B1R, suggesting a protective role of the former while a deleterious role of the latter in epileptogenesis (Adolfo Argañaraz et al., 2004). In another study, however, quite the opposite was reported where B1R knockout animals were found to be more susceptible to seizures through possible mechanisms where B2R could be a mediator of increased hippocampal excitability and susceptibility to seizures, suggesting B1R rather protective and B2R somewhat exacerbating toward the pathogenic profile (Rodi et al., 2013). Therefore, exploring the direct role of kininogen or bradykinin in epileptogenesis is crucial. To this end, in the current study, we carried out a brain-specific overexpression of kininogen and injection of bradykinin, and investigated their influences on seizure occurrence.

## 2 Materials and methods

### 2.1 Animals

Animal experiments were conducted at both Shanghai Jiao Tong University (SJTU) and the Indian Institute of Technology Kharagpur (IIT-KGP), and ethical approval was obtained from relevant committees of both parties. All mice (both wild-type [WT] and transgenic) were of C57BL/6J background, and only males were used to avoid any confounding effects arising from hormonal changes in females during different phases of an estrous cycle (Dhir, 2012). Surgeries and related treatments were started at such a time that the final target studies could be carried out by postnatal day 60 (P60) for studying adult-life epileptogenesis.

All kininogen-related studies at SJTU used WT mice bought from Charles River (Zhejiang, CN) at least 1 week before study commencement and kept for the rest of the studies at the Laboratory Animal Center of SJTU. WT mice used for bradykinin-related seizure susceptibility studies, and Thy1-GCaMP6f and PV^Cre^-GCaMP6f mice used for bradykinin-related calcium imaging studies conducted at IIT-KGP were purchased from the Jackson Laboratory. Thy1-GCaMP6f mice were the result of backcrossing Thy1-GCaMP6f males (JAX # 024276) with WT females, while PV^Cre^-GCaMP6f mice were the result of backcrossing the PV^Cre^ (JAX # 008069) males with Ai95 (RCL-GCaMP6f)-D (JAX # 024105) females. All animals were housed under standard laboratory conditions with unrestricted food and water, and maximal effort was made to ensure minimal pain and discomfort, and to minimize the number of animals used in the study. A light-dark cycle of 12-h/12-h was maintained (light on at 08:00 and light off at 20:00), and all the seizure susceptibility experiments were performed in between 12:00-18:00 with a light intensity of ∼300 lux.

### 2.2 Single-cell RNA-sequencing (scRNA-seq) datasets and bioinformatic analyses

One scRNA-seq dataset containing information on the mouse brain was obtained from Zenodo (Record # 8041114 & 8041323) and Single Cell Portal (SCP) (Study # SCP1835), which consisted of 5,413 genes from adult mouse brain tissues, yielding a spatial cell atlas of 119,173 cells (Zeng et al., 2023). Another scRNA-seq data of 3.5-month-old human whole-brain organoid (the male GM08330 iPSC line) was obtained from SCP (Study# SCP1756) (Ana, 2022). The analysis of scRNA-seq data was performed as described previously (Thakur et al., 2023). Briefly, they were analyzed with R (v4.4.2). The following packages were used for loading, saving, and manipulating data, as well as data integration, analysis, and generating plots: Seurat (v4.3.0), tidyverse (v1.3.2), dplyr (v1.1.0), patchwork (v1.1.2), stringr (v1.5.0), Harmony (v0.1.1), org.Hs.eg.db (v3.16.0), and SingleR (v2.0.0). The read count matrix was read into R, creating the Seurat object with the CreateSeuratObject() function. The t-distributed stochastic neighbor embedding (tSNE) maps were generated using the runTSNE() function, with the first 20 dimensions. Violin plots were generated using the VlnPlot() function. Broad Institute’s single-cell portal was also utilized for data analyses.

### 2.3 Mouse brain sample collection and western blotting

After cardiac perfusion with ice-cold phosphate buffered saline (PBS), mouse brains were taken out, and both hippocampal and cortical samples were separated and snap-frozen in liquid nitrogen, followed by storage at −80°C. On the day of protein extraction, tissues were homogenized in Radio Immuno-Precipitation Assay buffer with added protease inhibitors on an automatic homogenizer, followed by centrifuging at 12,000 g for 30 min at 4°C (Zou et al., 2019). The supernatant was collected, and bicinchoninic acid assay was done to quantify the protein concentrations. For protein separation, 15 μL of lysates containing 30 μg of protein was run per lane on a 7.5% sodium dodecyl sulfate–polyacrylamide gel electrophoresis gel. After separation, proteins were transferred onto a 0.45-μm polyvinylidene difluoride membrane and blocked in 5% bovine serum albumin (BSA) for 1.5 h at room temperature. The membranes were then incubated overnight in primary antibodies containing 5% BSA and incubated in secondary antibodies in 5% BSA on the next day after 3 × 5 min washes in Tween 20-containing Tris-Buffered Saline. After the final wash following secondary antibody incubation, the target proteins were visualized with a 1:1 mixture of a peroxide solution and a luminol enhancer solution, and images were obtained using a Bio-Rad ChemiDoc Imaging System. The following antibodies were used: Rabbit Anti-Kininogen1 (Abcam ab175386, 1:1000), Rabbit Anti-GAPDH (KeyGEN Biotech, Jiangsu, CN, 1:10000), and Goat Anti-Rabbit IgG H&L (HRP) (Abcam ab6721, 1:10000).

### 2.4 Immunofluorescence sample processing and confocal imaging

After cardiac perfusion with ice-cold PBS and 4% paraformaldehyde (PFA), mouse brains were immersed into 4% PFA for another 18–24 h, and then submerged into graded (20%, and 30%) sucrose solutions. Next, the brains were embedded onto optimal cutting temperature compound, and sliced (30-μm) on a Leica CM3050S cryostat. After mounting, the slices were stored at −80°C until use. Immunostaining was done by standard procedures, including a 90-min blocking in 5% normal goat serum in 0.3% tween 20-containing PBS, followed by overnight incubation in primary antibodies and incubation for 1 h with fluorophore-conjugated secondary antibodies on the following day. Finally, slices were preserved using a DAPI (Beyotime) mounting solution and imaged on a Leica TCS SP8 confocal microscope. The following antibodies were used: Rabbit Anti-Kininogen1 (Abcam ab175386, 1:1000), Mouse Anti-NeuN (Invitrogen MA5-33103, 1:800), Mouse Anti-Iba1 (Invitrogen MA5-27726, 1:500), Anti-GFAP (Invitrogen 14-9892-82, 1:1500), Alexa Fluor™ 488 Goat Anti-Rabbit (Invitrogen), Alexa Fluor™ 594 Goat Anti-Rabbit (Invitrogen), Alexa Fluor™ 488 Goat Anti-Mouse IgG (Invitrogen), and Alexa Fluor™ 594 Goat Anti-Mouse (Invitrogen).

### 2.5 Pilocarpine-induced status epilepticus (Pilo-SE)

After 30 min of injection with an atropine solution (Sigma A0132, dissolved in physiological saline, 5 mg/kg, i.p.) to reduce peripheral effects, a single dose (300 mg/kg, i.p.) of pilocarpine (Sigma P6503) in 0.9% physiological saline was administered. Continuous Grade IV/V seizures occurring for 30 min was defined as SE (Zou et al., 2019), while Grade IV was defined as bilateral forelimb clonus and Grade V was defined as generalized tonic-clonic seizures with rearing and falling according to a modified Racine scale (Shapiro et al., 2019; Karlócai et al., 2011). After 30 min, SE was ceased by a chloral hydrate injection (BBI CB0288, Shanghai, CN, in physiological saline, i.p., 150 mg/kg), and extensive post-SE care was given to the animals to ensure maximal survival. Mice with SE were sacrificed after 5 days, and brains were collected for biochemical characterizations [5-day post-SE was chosen to overcome the 3-day latent phase (Karlócai et al., 2011), and ensure a possibility of the effects seen being due to a course of epileptogenic changes rather than just a mere acute response effect].

### 2.6 Plasmid construction, surgeries, and injections for kininogen overexpression

Plasmids for *AAV-Kng1* and its empty control (*AAV-Ctrl*) bearing an enhanced green fluorescence protein (*Egfp*) moiety were constructed by WZ Biosciences (Shanghai, CN). The following primers were used for *mKng1*: ATC​TAC​CTA​GGG​ATG​GAT​CCA​TGA​AGC​TCA​TTA​CTA​CAC​T (F) and CTT​CCT​CTG​CCC​TCG​AAT​TCT​TAA​GAA​AGA​GCA​TCA​AGG​A (R). Surgeries were performed under isoflurane anesthesia, and the AAVs were injected into the dorsal hippocampal CA1 area (from Bregma, AP: –2.00 mm, ML: ±1.50 mm, DV: –1.30 mm) by a 10-μL syringe and a 33-gauge needle (Hamilton) at a speed of 0.1 μL/min under 1%–1.5% isoflurane anesthesia following a brief initial induction period at 3%. The syringe was left in place for 15 min for complete diffusion of the AAV. A 4-week gap was given for a satisfactory overexpression of kininogen before going for the downstream studies. Overexpression was confirmed by western blot, while the injection site accuracy was checked by fluorescence imaging of EGFP.

### 2.7 Surgeries, implants, and injections for bradykinin-related experiments

For all bradykinin-related studies (behavior and calcium imaging), a guide cannula of 1 cm was inserted at 6 mm posterior to bregma to a depth of 3.50 mm [just above the fourth ventricle (4V)]. For calcium imaging, however, an additional imaging window consisting of two 3-mm and a 5-mm cover glass (Warner Instruments, 64–0720 and 64–0700, respectively) was implanted, as described earlier (Agarwalla et al., 2023), over the primary motor cortex area (from Bregma, AP: +1.00 mm, ML: –2.00 mm, according to the Paxinos mouse brain atlas) of the left hemisphere (this hemisphere was chosen due to specific imaging setup of the laboratory). After surgery, C&B Metabond (Sun Medical, Japan) was applied around the guide cannula and the imaging window for stable implants, with additional supplementation with common dental cement, where needed. All standard post-surgical care were given for at least 3 days, and the following treatments/experiments were started at least after 7 days of recovery.

For bradykinin-related behavioral studies, after a 7-day rest post-surgery, varying doses of bradykinin in acetate form (Sigma B3259; 0.1 μM, 1 μM, and 10 μM; dissolved in sterile PBS) were injected into the 4V (from Bregma, AP: −6.00 mm, DV: −4.00 mm) through the implanted guide cannula. Each group had 9–10 animals, including a sex- and age-matched PBS-Ctrl group (injected with only PBS, devoid of bradykinin). A total volume of 2 μL of PBS or bradykinin solution was injected at a rate of ∼0.33 μL/min. The injections were repeated for 7 days (once/day at the same time every day), and were done with a 5-μL Hamilton syringe and a 26s-gauge needle. The needle was left in place for 3 min post-injection to ensure complete diffusion of the injectable. After the last day of injection, the animal was given a rest for 30 min to ensure adequate recovery from anesthesia before going for behavioral studies. For calcium imaging, all injection procedures were just the same, except that only one dose of bradykinin was chosen based on the outcome of the behavior study. All surgeries, implants, and 4V injections were done under 1%–1.5% isoflurane anesthesia following a brief initial induction period at 3%.

### 2.8 Pentylenetetrazole-induced seizure susceptibility test (PTZ-SST)

For PTZ-SST, repeated PTZ (Sigma P6500, in sterile 0.9% physiological saline, i.p.) shots were administered until the animals displayed Grade V seizures (Supplementary Video S1). For kininogen-PTZ-SST, the starting dose of PTZ was 40 mg/kg, followed by two 5 mg/kg doses 10 min apart and then 10 mg/kg doses every 5 min until Grade V seizures were evoked. For bradykinin-PTZ-SST, the timeline for PTZ administration was the same except for the first dose, which was 30 mg/kg - decided to be lowered due to previous observations. If the Grade V seizures continued for more than 5 min, the animals were given Diazepam (1 mg/kg, i.p.) to prevent seizure-related deaths. The cumulative dose to evoke Grade V seizures was calculated and compared among groups.

### 2.9 Bradykinin-related 2-photon calcium imaging

Upon completion of PTZ-SST, one dose (0.1 μM) of bradykinin was chosen based on the experimental outcome and was used further for calcium imaging studies. For excitatory neuron imaging, a total of six Thy1-GCaMP6f male mice were divided into two groups (BDK + PTZ: 3, and PBS + PTZ: 3). For inhibitory neuron imaging, similarly, a total of six PV^Cre^-GCaMP6f male mice were divided into two groups. After a rest of 7-day post-implant surgery (Section 2.7), the animals were habituated to stay on the imaging platform for 15–60 min every day for a total of 7 days (gradually increasing the time from 15-min on day one to 60-min on day seven) to facilitate awake imaging of the animals. After this period, all the animals were habituated to staying calm on the imaging platform in a head-fixed yet awake and calm manner. When the habituation was complete, the animals were administered (4V) with the said dose of bradykinin or PBS for 7 days (similar to the bradykinin-PTZ-SST, but with the 0.1 μM dose only). All the animals were kept on the imaging platform, even on the injection days, to prevent reversal of the habituation. After the last day of bradykinin injection, the animals were given a rest for 30 min to ensure adequate recovery from anesthesia (similar to the bradykinin-PTZ-SST). Then, a single subthreshold dose of PTZ (30 mg/kg) was given to study the neuronal calcium activity without causing full-blown convulsions (which was not only due to technical limitations of seizure imaging but also beyond this study’s aim). Cortical L2/3 neurons (both excitatory and inhibitory, from Thy1-GCaMP6f and PV^Cre^-GCaMP6f mouse strains, respectively, containing genetically encoded green fluorescence protein calcium indicator [GCaMP6f]) dispersed through the depth of 180–250 μm (from the dura surface) were visualized with a 20×/0.8 NA water-immersion objective (Olympus, Japan) using a 920-nm Insight (Spectra-Physics) laser (50–80 mW power). The signal was further amplified by photo-multiplier tubes using Bruker’s 2-photon microscopy system controlled with the Prairie View software (v5.3U2beta) (Mehra et al., 2022). An ROI of 240 × 144 pixels, 1.183 μm/pixel at a 512 × 512 resolution, was used for all imaging. A pixel dwell time of 5.6 μs was used, leading to a frame duration of ∼249 ms (∼4 fps). Unlike PTZ-SST, the calcium imaging studies were conducted inside a dark chamber, as because the photo-multiplier tube of the imaging setup is light-sensitive.

### 2.10 Calcium imaging data preprocessing and analysis

Images of each frame (∼249 ms) for every 5-minute-long session were read into MATLAB, and motion correction in the x-y or imaging plane was performed (Agarwalla et al., 2023; Mehra et al., 2022; Bandyopadhyay et al., 2010). Cells were manually marked based on the average of 1205 motion-corrected frames (1205 * 0.249 ∼ 300 s) for each 5-min period. The luminance of each cell (say *i*th) in each frame (say *j*th) was calculated based on all the pixels inside a 5-pixel-radius circle, considering the manually selected point as the center of the cell (*F*_*ij*_). The cells’ relative fluorescence, *ΔF/F*, time series were calculated as (*F*_*ij*_-*F*_*i0*_)/*F*_*i0*_, where *F*_*i0*_ is the median fluorescence of the cell over all frames. Since the baseline can fluctuate throughout imaging within each 5-min session, *ΔF/F* could fluctuate below baseline (median) and does not reflect hyperpolarization. Events in the traces were detected based on smoothing over 5 frames and finding a threshold crossing of 1.96 STD of the *ΔF/F* of each cell. Local *ΔF/F* traces of events were obtained for each event by considering a baseline of 8 frames, ∼2-s, and ensuring calcium transient by considering the baseline to be always within 1 STD, which allows apparent onset to be detected after a 2-s flat baseline and reduces spurious detection. Finally, the mean of the trace over 3-s was checked for significant differences (*p < 0.05*) compared to the mean of baseline. The number of events detected in each cell per 5-min window was used for further statistical analyses.

### 2.11 Statistical analyses

Statistical analyses were done with both MATLAB and GraphPad Prism v9.0.0, while graphs were made using GraphPad Prism v9.0.0. Normality was checked using the Shapiro–Wilk test. A two-tailed unpaired t-test was performed to compare between two groups with normal distributions with non-significant differences in their standard deviation (SD). Two groups with normal distributions but with significant differences in their SD were analyzed with two-tailed t-test with Welch correction. Two groups with non-normal distributions were tested using the Mann-Whitney U test. Tests with more than two groups containing variables with non-normal distributions were tested using the Kruskal–Wallis test. Calcium images and traces were processed, and analyzed in MATLAB using a custom-written software (Agarwalla et al., 2023; Mehra et al., 2022; Bandyopadhyay et al., 2010). A two-way ANOVA was used for comparisons between the groups with multiple timepoints for calcium imaging (with groups as the ‘between-subjects’ factor, and the timepoints as ‘within-subjects’ factor). For statistical comparisons of all data, *p < 0.05* was considered statistically significant (*). Effect size was quantified as Cohen’s d (*d*) for t-tests, as rank-biserial correlation coefficient (*r*) for Mann-Whitney U tests, and as partial eta square (*η* ^*2*^) for ANOVA tests.

## 3 Results

### 3.1 Bioinformatic analyses of scRNA-seq datasets revealed genetic signatures of *mKng1/hKNG1* in the brain

**FIGURE 1:**
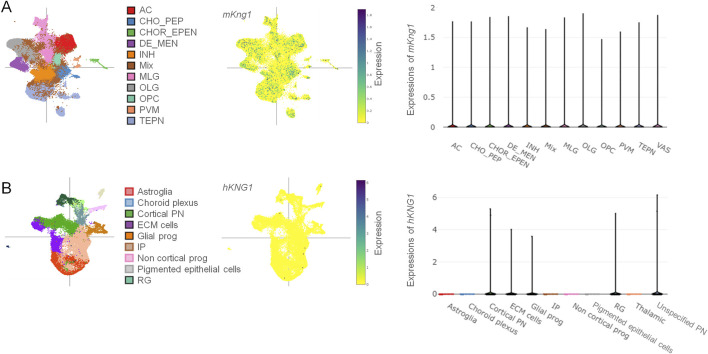
Single-cell RNA sequencing data reveals the presence of *mKng1/hKNG1* mRNA among various CNS cell types. **(A)** Different cell types were studied from the scRNA-Seq dataset obtained from mouse brain samples (*left*); tSNE plot of cell-type specific distribution pattern of m*Kng1* in mouse brain samples (*middle*); violin plot of cell-type specific expression levels of m*Kng1* in mouse brain samples (*right*). **(B)** Different cell types were studied from the scRNA-Seq dataset obtained from developing human cerebral cortex organoid (*left*); tSNE plot of cell-type specific distribution pattern of *hKNG1* in developing human cerebral cortex organoid (*middle*); violin plot of cell-type specific expression levels of *hKNG1* in developing human cerebral cortex organoid (*right*). Abbreviations: AC, astrocytes; CHO_PEP, cholinergic, monoaminergic and peptidergic neurons; CHOR_EPEN, choroid epithelial cells and ependymal cells; DE_MEN, di- and mesencephalon neurons; INH, inhibitory neurons; MLG, microglia; OLG, oligodendrocytes; OPC, oligodendrocytes precursor cell; PVM, perivascular macrophages; TEPN, telencephalon projecting neurons; VAS, vascular cells; ECM, extracellular matrix; IP, intermediate progenitor; PN, projecting neurons; RG, radial glia.

Kininogen is known to be synthesized primarily in the liver and expressed in many other organs and cell types (Lalmanach et al., 2010). However, its brain expression data is scarce and requires much-warranted validation. To this end, we first performed scRNA-seq data analyses from datasets publicly available online, both for *mKng1*, from an adult mouse brain dataset (Zeng et al., 2023), and for *hKNG1*, from a developing human cortical organoid dataset (Ana, 2022). Data from the mouse brain showed that *mKng1* mRNA is present in various cell types of major importance, including neurons, astrocytes, microglia, and oligodendrocytes. Moreover, it was found in the oligodendrocyte precursor cells, choroid epithelial and ependymal cells, vascular cells, and perivascular macrophages (Figure 1A). A variety of different neuronal subtypes—including cholinergic, monoaminergic, and peptidergic neurons, as well as neurons projecting from different brain regions (diencephalon, mesencephalon, and telencephalon) and inhibitory interneurons—all showed expression of *mKng1* (Figure 1A). Similarly, data from the developing human cerebral cortex organoid revealed that *hKNG1* is also present in the developing brain organoids with a very high presence in the cortical projection neurons, glial progenitor cells, radial glia, and extracellular matrix cells (Figure 1B).

### 3.2 Kininogen is basally and preferentially localized in neurons but not in glia

**FIGURE 2:**
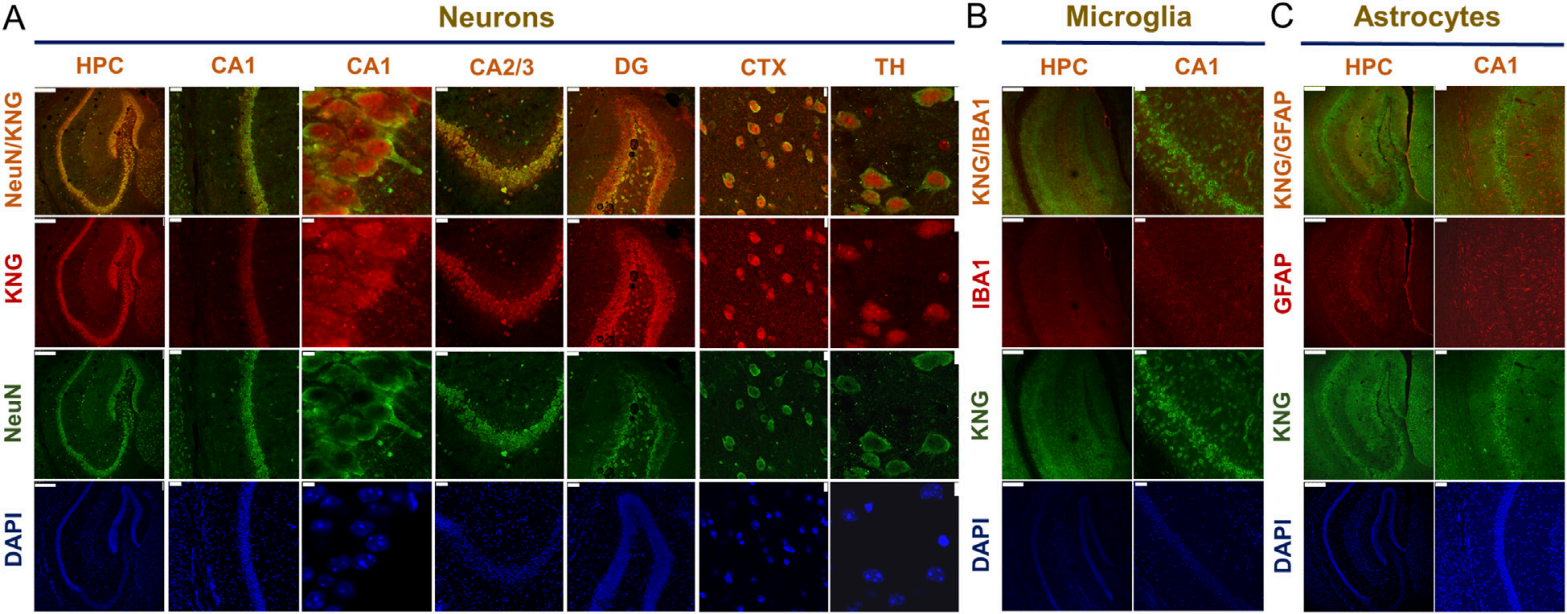
Kininogen is predominantly localized in neurons but not in glia. **(A)** Immunofluorescent co-staining of NeuN and KNG in the hippocampus and hippocampal subregions (CA1/CA2/CA3/DG), cortex, and thalamus. Scale bars: 250 μm for Column I, 50 μm for Column II/IV/V, 5 μm for Column III; 10 μm for Column VI, and 7.5 μm for Column VII (from left to right). **(B)** Immunofluorescent co-staining of KNG and IBA1 in the hippocampus and CA1. Scale bars: 250 μm for Column I, 50 μm for Column II (from left to right). **(C)** Immunofluorescent co-staining of KNG and GFAP in the hippocampus and CA1. Scale bars: 250 μm for Column I, 50 μm for Column II (from left to right). Abbreviations: KNG, kininogen; HPC, hippocampus; CA, Cornu Ammonis; DG, dentate gyri; CTX, cortex; TH, thalamus.

By employing double immunofluorescence staining on slices from Naïve (untreated) WT mouse brains, we checked the expression profiles of kininogen in neurons and glial cells among several brain regions. In the hippocampus, cortex, and thalamus, kininogen was found to be expressed in neurons (Figure 2A). However, neither microglial cells (Figure 2B) nor the astrocytes (Figure 2C) in the hippocampal area expressed kininogen. A similar trend was found among the cortical and thalamic glial cells, which did not express kininogen (data not shown), suggesting a preferential localization of kininogen in neurons rather having ubiquitous expressions over all the cell types.

### 3.3 Changes in kininogen levels and cHK to iHK ratios are evident in the mouse hippocampus in the early stages of status epilepticus

**FIGURE 3:**
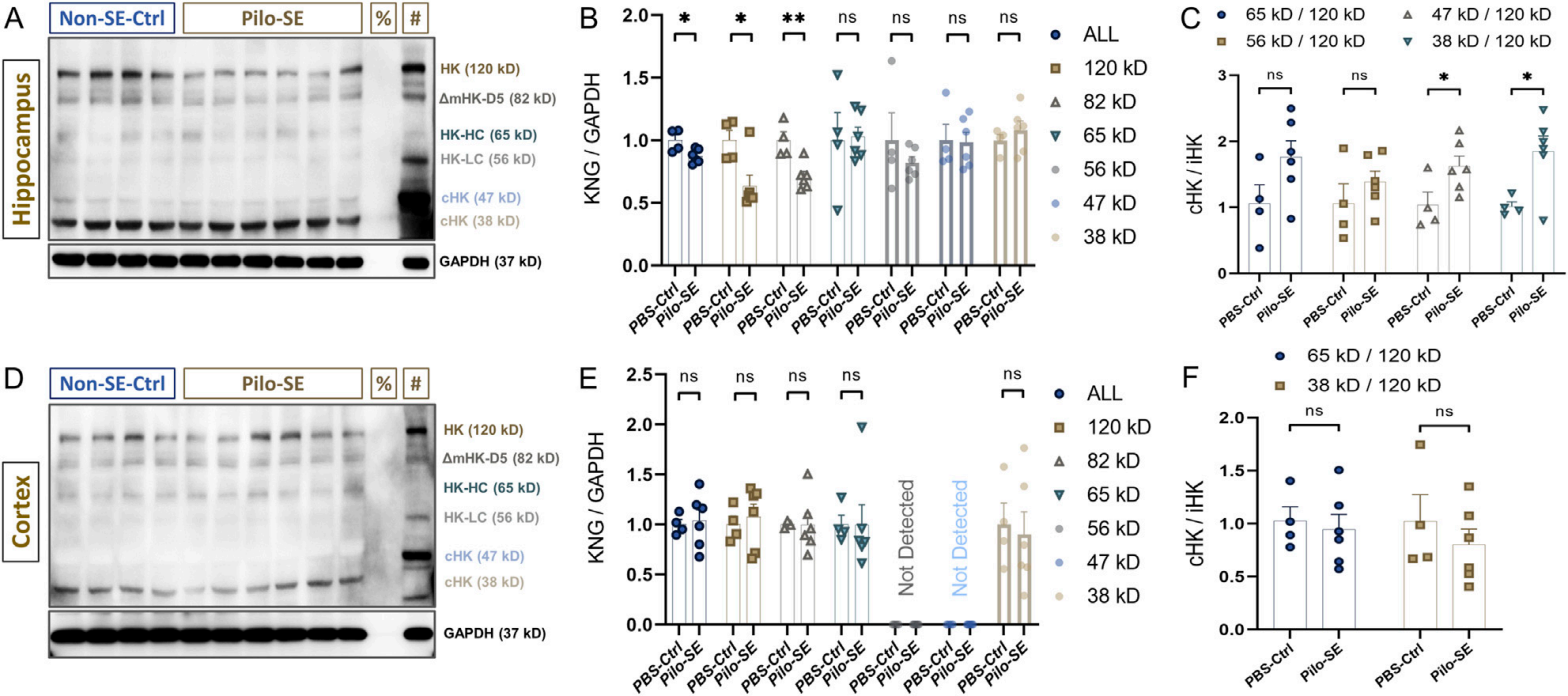
Kininogen dynamics in the hippocampus and cortex of Pilo-SE mice. **(A)** Hippocampal western blot bands of kininogen [^%^Negative control (Albumin); ^#^Positive Control (Untreated mouse liver)]. **(B)** Expression levels of total kininogen and its subtypes in the hippocampus after Pilo-SE. **(C)** cHK to iHK ratio in the Pilo-SE hippocampus. **(D)** Cortical western blot bands of kininogen [^%^Negative control (Albumin); ^#^Positive Control (Untreated mouse liver)]. **(E)** Expression levels of total kininogen and its subtypes in the cortex after Pilo-SE. **(F)** cHK to iHK ratio in the Pilo-SE cortex. Abbreviations: KNG, kininogen; Pilo-SE, Pilocarpine-induced status epilepticus; cHK, cleaved high-molecular weight kininogen; iHK, intact high-molecular weight kininogen.

Next, we investigated whether Pilo-SE could alter the kininogen levels in the hippocampus and cortex. Except for the 120-kD iHK and the 37-kD cHK subtypes, the bands for the other subtypes were very dull but somewhat visible (Yamamoto-Imoto et al., 2018) across both the test groups (Figure 3A). Bands across the following positions were found: ∼120 kD, ∼82 kD, ∼65 kD, ∼56 kD, ∼47 kD, and ∼38 kD (Figure 3A). Densitometric analyses of all the bands revealed that Pilo-SE animals (*n = 6*) having slight but statistically significant reductions in summed expression of all kininogen isoforms (*ΔMean ± SEM: −0.125 ± 0.0451, p = 0.024 by unpaired two-tailed t-test (d = 1.62)*; Figure 3B) in the hippocampus, when compared to the Controls (*n = 4*). And, that of the 120-kD iHK (*ΔMean ± SEM: −0.365 ± 0.126, p = 0.038 by Mann-Whitney test (r = -0.83)*; Figure 3B) and 82-kD ΔmHK-D5 (*ΔMean ± SEM: −0.2895 ± 0.07519, p = 0.0049 by unpaired two-tailed t-test (d = 2.24)*; Figure 3B) were also decreased significantly (44.5 % and 29%, respectively). The 65-kD band that represents both the LK and the heavy chain of HK remained unchanged (*ΔMean ± SEM: 0.03043 ± 0.1997, p = 0.8827 by unpaired two-tailed t-test*; Figure 3B). A 56-kD band that represents the light-chain of HK (HK-LC) also remained unchanged (*ΔMean ± SEM: 0.1771 ± 0.2259, p = 0.4859 by unpaired two-tailed Welch test*; Figure 3B). The 47-kD cHK band also showed no intergroup differences (*ΔMean ± SEM: −0.01484 ± 0.1441, p = 0.9143 by Mann-W. test*; Figure 3B). There was another degradation product of kininogen detected at 38-kD which also showed no change in the Pilo-SE group compared to the Control animals (*ΔMean ± SEM: 0.08285 ± 0.09801, p = 0.4225 by unpaired two-tailed t-test*; Figure 3B). The ratio changes of these degradation products of 47 kD and 38 kD to iHK in the hippocampus, however, were significantly higher (*ΔMean ± SEM: 0.5848 ± 0.2501, p = 0.0475 by unpaired two-tailed t-test for 47-kD* vs*. 120-kD ratio (∼58.5%) (d = -1.36)*, Figure 3C; *and ΔMean ± SEM: 0.8366 ± 0.2943, p = 0.0217 by unpaired two-tailed t-test for 38-kD* vs*. 120-kD ratio (83.7%) (d = -1.66)*; Figure 3C). Ratio of the degradation products of ∼65-kD and ∼56-kD although showed similar pattern but did not reach a statistically significant threshold (70.08% changes, *p = 0.1001 for 65-kD*, and 32.9% changes, *p = 0.3227 for 56-kD*). In the cortex, in contrast, neither any individual subtype nor the ratios of the cHK vs. iHK were found to be affected (Figures 3D–F).

### 3.4 Adeno-associated virus (AAV)-mediated overexpression of kininogen in the hippocampal CA1 region increases seizure susceptibility

**FIGURE 4:**
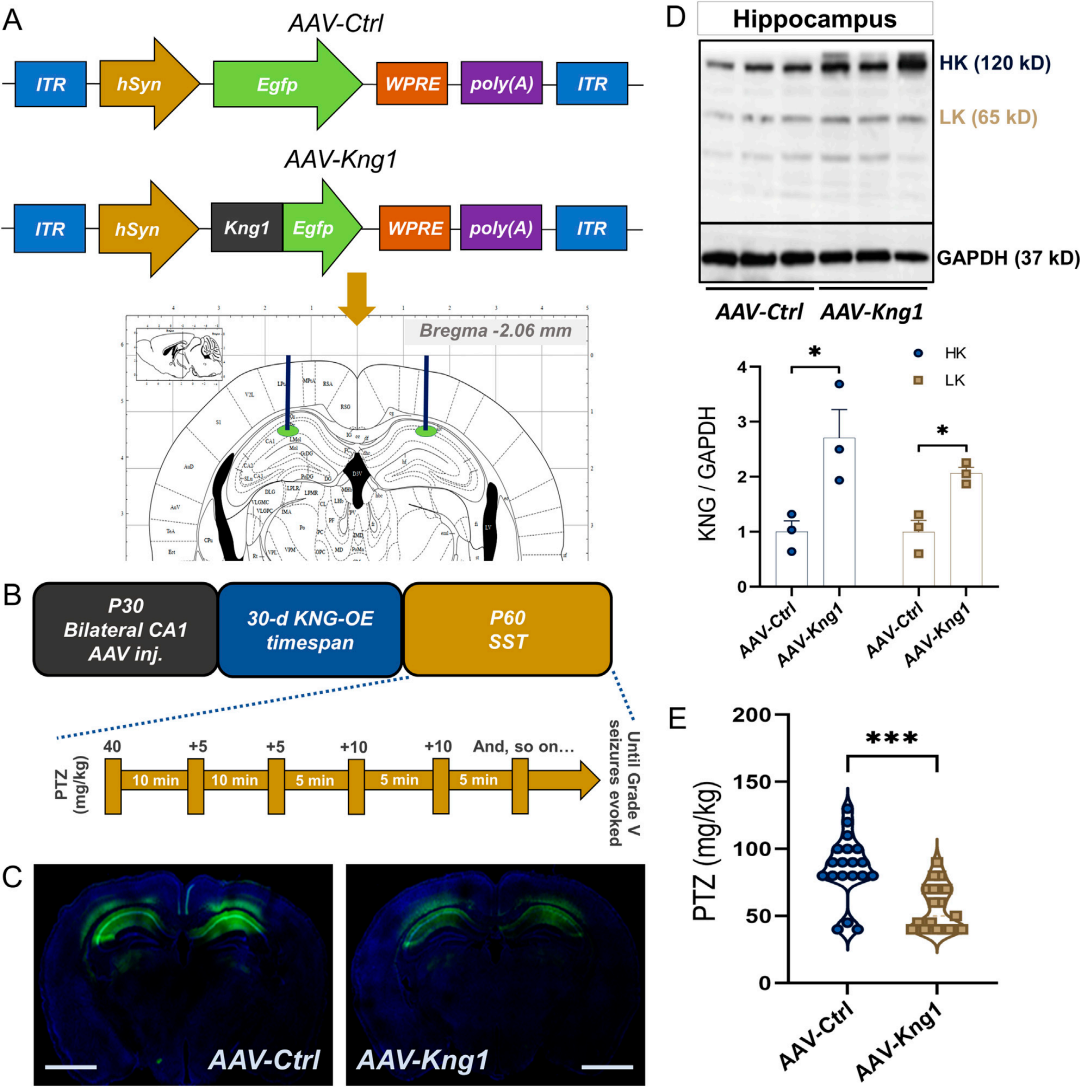
AAV-mediated *Kng1* gene delivery into the hippocampus increases seizure susceptibility in mice. **(A)** Structure of the *AAV-Ctrl* and *AAV-Kng1* plasmids and injection area on the Paxinos stereotaxic coordinates. **(B)** The timeline of AAV injection and seizure susceptibility test and PTZ injections. **(C)** CA1-specific injection site validation by EGFP fluorescence; Scale bar: 1.75 mm. **(D)** western blot bands (*top*) and quantification (*bottom*) of kininogen overexpression after AAV-mediated gene delivery into the hippocampal CA1 region. **(E)** PTZ-induced Grade V seizure threshold in mice with or without kininogen overexpression. Abbreviations: AAV, adeno-associated virus; CA, Cornu Ammonis; EGFP, enhanced green fluorescence protein; KNG, kininogen; OE, overexpression; PTZ, pentylenetetrazole; SST, seizure susceptibility test.

Next, to check whether such changes in the hippocampal levels of kininogen could be a causative factor for epileptogenesis, we microinjected *AAV-Kng1* encoding for a neuron-specific *hSyn* promoter and *Egfp* into the dorsal CA1 of WT mice (Figure 4A). A PTZ-SST was conducted 30 days after the AAV injection (Figure 4B). We confirmed the injection site through the fluorescence of translated EGFP (Figure 4C). The western blot results showed that both HK and LK increased by ∼2.0-fold (Figure 4D). The susceptibility experiments showed that CA1-specific overexpression of kininogen (n = 19) markedly reduces the cumulative dose of PTZ to evoke Grade V seizures compared to the Control mice (n = 19) (34% changes*, ΔMean ± SEM: −29.21 ± 6.762, p = 0.0001 by unpaired Mann-W. test (r = −0.69)*; Figure 4E), indicating a possible role of brain-localized synthesis of and/or activity on kininogen at early stages of brain injuries in rendering seizures.

### 3.5 Repeated bradykinin administration reduces seizure threshold

**FIGURE 5:**
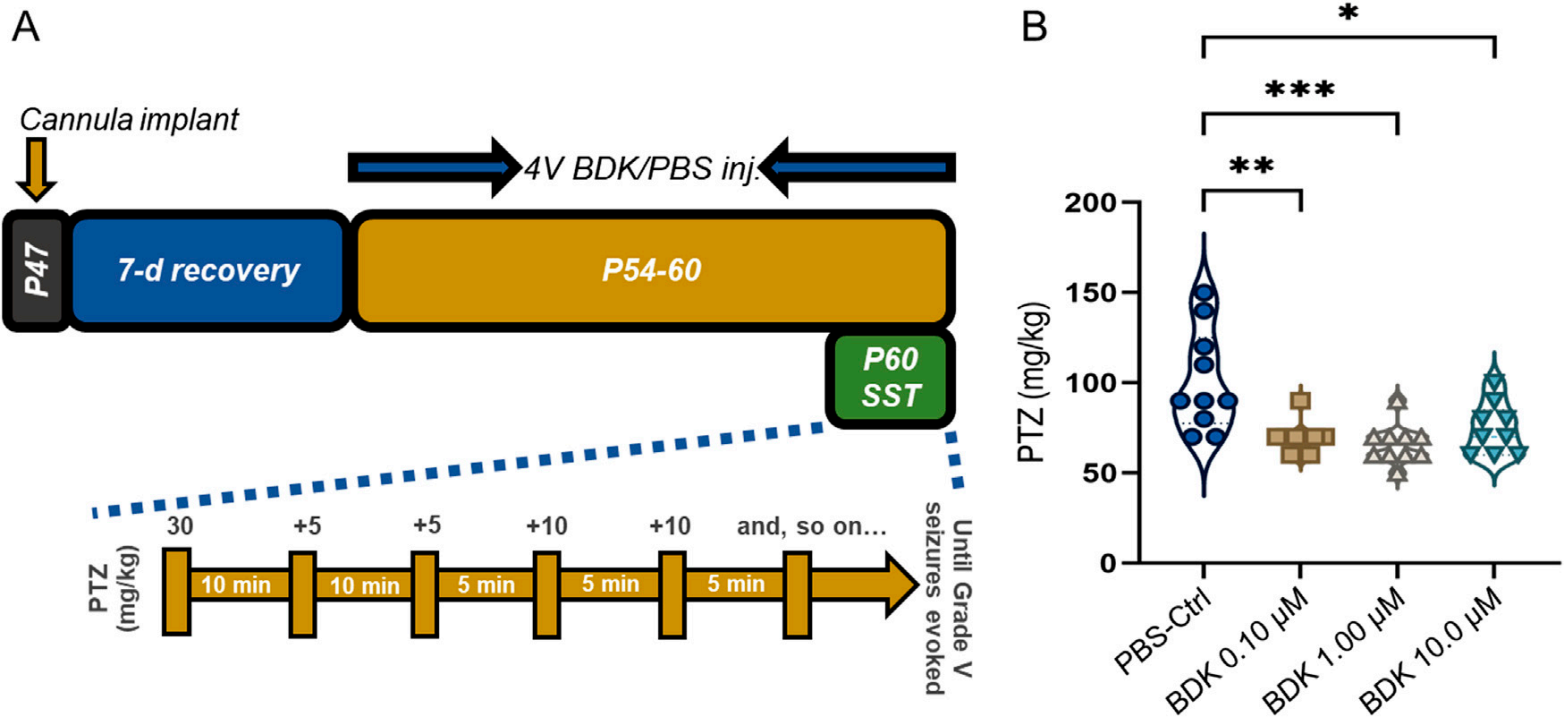
Bradykinin increases PTZ-induced seizure susceptibility in mice. **(A)** The timeline of the study. **(B)** PTZ-induced Grade V seizure thresholds in mice treated with PBS or differing doses of BDK. Abbreviations: BDK, bradykinin; PBS, phosphate-buffered saline; PTZ, pentylenetetrazole; and SST, seizure susceptibility test.

Bradykinin is highly expressed in the temporal lobe epilepsy (TLE) hippocampus (Simões et al., 2019). Combining our findings that overexpression of kininogen enhances seizure susceptibility, it is highly likely that, as a cleavage product of HK (Altamura et al., 1999), bradykinin possibly contributes to the effects of kininogen in reducing seizure onsets. To test this, we repeatedly injected three doses of bradykinin through the fourth ventricle for seven consecutive days and carried out a susceptibility test 30 min after the last bradykinin administration on the seventh day (Figure 5A). The starting dose of PTZ was reduced to 30 mg/kg because some animals exhibited Grade V seizures just after the first injection of 40 mg/kg in the previous set of experiments (Figure 4E). Bradykinin at all the doses significantly reduced the seizure threshold to evoke Grade V seizures (*ΔMean ± SEM: −32.00 ± 9.298, p = 0.0029 for 0.*1 μM (n = 10); *ΔMean ± SEM: −35.00 ± 9.504, p = 0.0004 for* 1 μM (n = 10), and *ΔMean ± SEM: −26.56 ± 10.40, p = 0.0271 for 10* μM (n = 9), vs*. PBS-Ctrl (n = 10) by unpaired Kruskal–Wallis test*; *F (3, 35) = 8.547, and η* ^*2*^ *= 0.39*] (Figure 5B).

### 3.6 Bradykinin exerts differential effects on excitatory and inhibitory neurons

**FIGURE 6:**
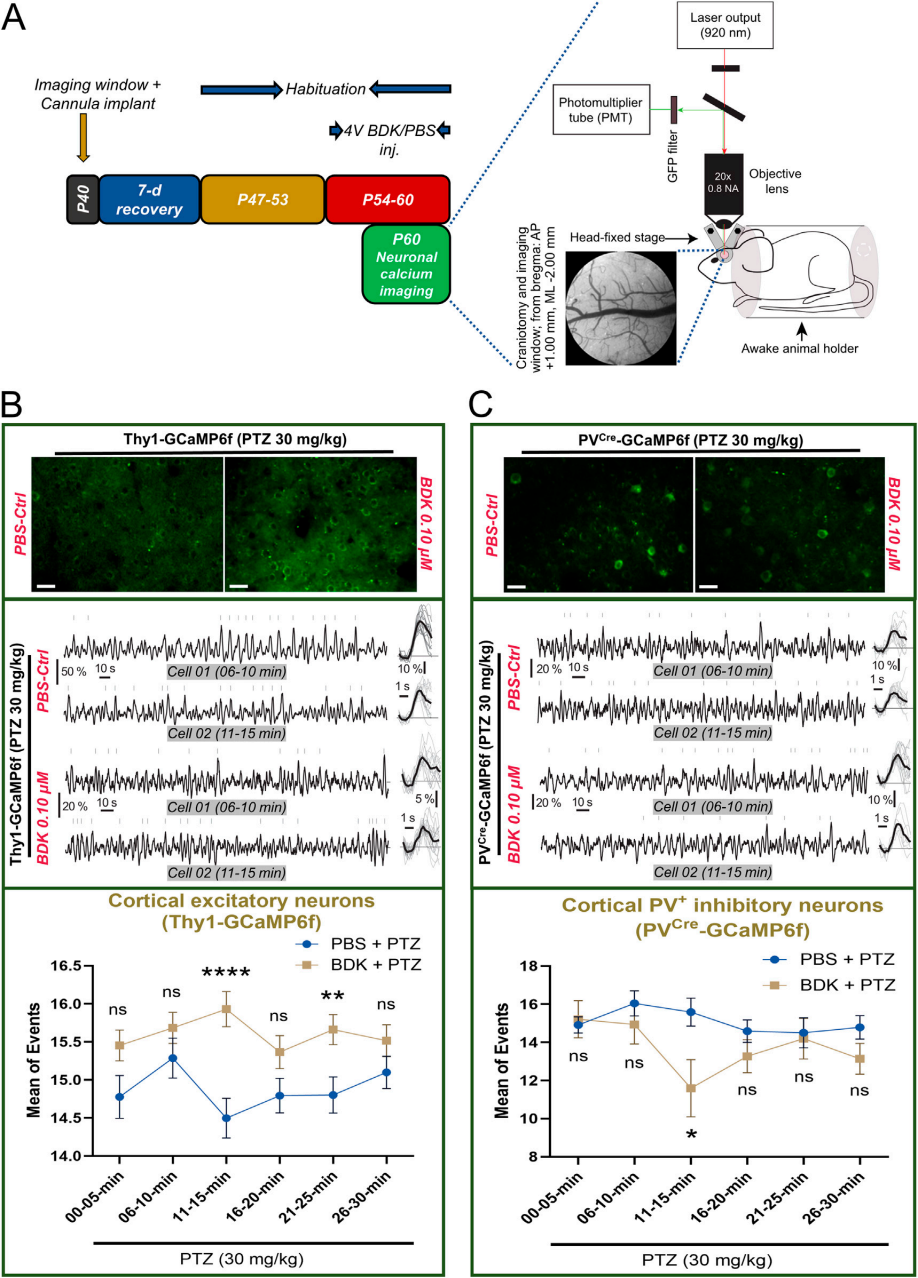
Bradykinin alters PTZ-induced calcium spikes in transgenic mice. **(A)** The timeline (*left*) and the setup (*right*) of the calcium imaging experiment; Scale bar, 0.5 mm for cranial window image. **(B)** (*Upper*) Representative images of GCaMP6f-tagged L2/3 cortical excitatory neurons from one animal from each group (average of 1205 frames from the 11–15 min timeline after PTZ treatment), Scale bar: 25 μm; (*middle*) Representative traces from two different time points from two different L2/3 cortical excitatory neurons; (*bottom*) and the % changes in the mean number of calcium events from L2/3 cortical excitatory neurons of the motor cortex after PTZ treatment (BDK vs. PBS). **(C)** (*Upper*) Representative images of GCaMP6f-tagged L2/3 cortical parvalbumin^+^ inhibitory interneurons from one animal from each group (average of 1205 frames from the 11–15 min timeline after PTZ treatment), Scale bar: 25 μm; (*middle*) Representative traces from two different time points from two different L2/3 cortical parvalbumin^+^ inhibitory interneurons; (*bottom*) and the % changes in the mean number of calcium events from L2/3 cortical parvalbumin^+^ inhibitory interneurons of the motor cortex after PTZ treatment (BDK vs. PBS). Abbreviations: BDK, bradykinin; PBS, phosphate-buffered saline; PTZ, pentylenetetrazole.

In order to investigate the mechanisms of how bradykinin may affect seizure threshold we next employed *in vivo* two-photon calcium imaging of excitatory and inhibitory neurons from awake Thy1-GCaMP6f and PV^Cre^-GCaMP6f mice, respectively (Figure 6A). Because bradykinin at all the doses showed near similar effects in rendering increased seizure susceptibility (Figure 5B), we chose the minimal effective dose of 0.1 μM in this study. After intracerebroventricular injections of this dose for seven consecutive days, imaging was done from the motor cortex area (Figure 6A). Calcium activities induced by PTZ (30 mg/kg, i.p.) were observed in excitatory neurons from Thy1-GCaMP6f mice (Figure 6B, upper and middle panel). The results showed that the mean number of events is increased in the bradykinin-treated animals (n = 161 neurons from 3 animals) compared to the PBS-treated animals (n = 145 neurons from 3 animals) at all the time points analyzed, showing a maximal effect at the 11–15-min timepoint (*ΔMean ± SEM: 1.434 ± 0.3468, p < 0.0001 by a two-way ANOVA test, F (1, 304) = 21.28, η* ^*2*^ *= 0.15 for Groups, η* ^*2*^ *= 0.35 for Timepoints, and η* ^*2*^ *= 0.08 for Group × Timepoint;* Figure 6B, lower panel). On the other hand, imaging of bradykinin-treated PV^Cre^-GCaMP6f mice (n = 15 neurons from 3 animals) showed a reduced mean number of calcium events in PV^+^ neurons compared to the PBS-treated mice (n = 24 neurons from 3 animals), causing highest effects at the 11–15-min timepoint (*ΔMean ± SEM: 4.591 ± 1.562, p = 0.0269 by a two-way ANOVA test, F (1, 37) = 4.534, η* ^*2*^ *= 0.18 for Groups, η* ^*2*^ *= 0.42 for Timepoints, and η* ^*2*^ *= 0.11 for Group × Timepoint;* Figure 6C, lower panel). These results demonstrate that bradykinin disrupts the balance between excitation and inhibition by activating the excitatory neurons and suppressing the inhibitory neurons, thereby enhancing susceptibility to PTZ-induced epilepsy.

## 4 Discussion

This study is the first to explore the direct effects of localized overexpression of kininogen and its nonapeptide fragment bradykinin in the brain on seizure susceptibility. We discovered that kininogen lowers the threshold for PTZ-induced seizures, indicating a crucial role of kininogen in facilitating epileptogenesis through possible downstream release of bradykinin and thereby causing in an imbalance of glutamatergic and GABAergic neuronal firings.

### 4.1 Does brain-derived kininogen participate in epileptogenesis?

Kininogen is mainly an inflammatory protein synthesized in the liver and pumped into the peripheral circulation during systemic inflammation (Takano et al., 1999). Although it is also known to express in the lungs and the kidneys (Mann and Lingrel, 1991), its brain-associated expression data are scarce; some data though highlighted its existence and association with vasopressin in several hypothalamic areas (Richoux et al., 1992). Our previous study demonstrated enhanced kininogen protein levels in the hippocampus and cerebrospinal fluid of rats at the early stage of Pilo-SE (Zou et al., 2019). However, being majorly a plasma protein, it remains of curiosity whether such existence in the brain could be a result of influx from the periphery or could it be due to its local synthesis. Herein, analyses of the brain scRNA-seq datasets showed that both *mKng1* and *hKNG1* are indeed present in the neurons in several brain regions (Figures 1A,B), and immunofluorescence study found that kininogen protein is expressed in the mouse brain, specifically in the neurons (Figure 2A) but not in the glia (Figures 2B,C). This indicates that the brain-resident cells, specifically neurons may indeed be capable of synthesizing this protein; if not so, and if the observed expressions were due to peripheral influx, then the glial cells too possibly would have shown its expressions, which was not the case. Also, as some other data suggest, its mRNA in the developing human cortical organoid astrocytes is indeed absent (Figure 1B), and so is it in cultured rat astrocytes (Takano et al., 1999).

Despite our previous findings on the rising levels of kininogen in the Pilo-SE rat hippocampus (Zou et al., 2019), in the present study on mice, we found that not only the overall turnover of kininogen but also the iHK isoform of 120-kD were significantly reduced in the hippocampus of the Pilo-SE mice (Figure 3B). This discrepancy with previous results could be due to differential processing of the brain samples, where the samples in the current study were made blood-free and thus reducing any chance of kininogen contamination originating from the periphery, which was not the case for the previous study. Moreover, reporting of only one kininogen subtype in the previous study (Zou et al., 2019) has limited our understanding and comparison on the changes in all the molecular weight ranges found in the current study. Nevertheless, contamination of peripheral kininogen could be a big contributor to what results we had found in the previous study (Zou et al., 2019). Interestingly, apart from iHK, a marked reduction in the 82-kD was also noteworthy. The 82-kD subtype, although lacking the D5, also may contribute to bradykinin liberation as it still holds the D4, which is responsible for bradykinin release. In the current study, however, the cHK subtypes, including the 65-kD, 56-kD, 47-kD, or 38-kD were not changed despite a reduction seen in the 120-kD or the 82-kD band. Utilizing the levels of cHK as a biomarker for various diseases has been an emerging topic of interest (Yamamoto-Imoto et al., 2018; Suffritti et al., 2014; Sparkenbaugh et al., 2020). Depletion of iHK may in general be an indicator of high activity of the related KKS components, while releasing bradykinin and causing in an upregulation of the cHK subtypes, at least under certain experimental conditions (Henderson et al., 2021; Reddigari and Kaplan, 1988; Simões et al., 2019; Yamamoto-Imoto et al., 2018). However, that may not be the case always, especially in cases where the samples under the test are not influenced by further external stimuli such as the addition of an enzyme. Plasma levels of cHK in sickle cell disease (SCD) patients, for example, were found higher than their controls despite no changes found in iHK levels between these groups (Sparkenbaugh et al., 2020). Similarly, cHK subtypes were indifferent in our case despite a marked reduction in iHK levels. Such findings could signify possibilities where lowered iHK levels are possibly caused by its downregulated translation rather than its enzymatic degradation. In such cases where the cHK levels are still the same while the iHK levels are reduced, representation of the increased cHK to iHK ratio (cHK/iHK) could serve as an indicator of increases in iHK cleavage despite its reduced translation (Suffritti et al., 2014; Sparkenbaugh et al., 2020). To this end, we found that, at least in the hippocampus, the ratio of all the cHK subtypes to iHK was much higher than the Controls (Figure 3C). This could possibly mean that the enzymatic degradation of kininogen is still higher in the Pilo-SE group despite reduced translation of kininogen, and thus causing higher bradykinin liberation is this group of animals. Even if the bradykinin release would be the same in the Pilo-SE animals vs. the Control animals, owing to the overall vulnerability of an SE brain, in combination with other structural and molecular changes, similar levels of bradykinin release would probably cause much higher damages in an SE brain compared to a normal brain. Pilocarpine-induced SE and subsequent development of chronic seizures reflect many similar characteristics with the clinical TLE. Inflammation, among others, can be a crucial aspect of TLE patient brains; post-mortem hippocampus from TLE patients, for example, show heightened inflammatory features both at the cellular and molecular levels (Das et al., 2012). In animal models of Pilo-SE as well, upregulation of inflammatory markers in the hippocampus is evident (Wang et al., 2019). Changes in some of these inflammatory molecules have been found to be associated with kininogen in various other diseases, and hippocampal regulation of kininogen found in this study may well correlate with such inflammatory activities. Hippocampus is a significant brain area for epileptic seizure initiation that further spreads to the amygdala and to the neocortex (Wang et al., 2022; Turski et al., 1983a; Turski et al., 1983b). Herein, changes in kininogen levels in the hippocampus (Figures 3A–C) but not in the cortex (Figures 3D–F) suggest that the regulation of kininogen synthesis and/or degradation takes place in the resident brain cells (Dendorfer et al., 1997) of the seizure-initiation related areas such as the hippocampus that may be involved in the quickened onset of epilepsy, through direct or indirect means. Indeed, KKS activity, including plasma kallikrein activity, is heightened in the serum of patients with TLE (compared to healthy subjects), leading to breakdown of HK and releasing bradykinin (Simões et al., 2019). Therefore, considering the findings: 1) the presence of *mKng1* in mouse brain neurons; 2) Expression of kininogen in both hippocampal and cortical neurons; 3) Elevated activity of possible kininogen degradation and increased cHK/iHK ratio in the hippocampus of Pilo-SE mice, we conclude that in addition to the leakage of peripheral kininogen into the brain parenchyma (Simões et al., 2019), localized regulations of kininogen, at least in part, in the hippocampus may be a major contributor to epileptogenesis.

### 4.2 Increased seizure susceptibility: the role of kininogen and potential interacting factors

We found that CA1-specific overexpression of kininogen is sufficient to reduce the threshold of PTZ-induced Grade V seizures in mice (Figure 4E). Pyramidal neurons in the CA1 are known to be hyperactive and highly vulnerable in epileptic humans and rodent models of epilepsy (Hattiangady et al., 2011). Many studies have demonstrated a significant loss of CA1 neurons in epilepsy mouse models (Liu et al., 2017). A CA1-specific mRNA profiling showed that apart from the neuronal excitability-related gene products, immune-related gene products are also highly present in the CA1 area after an SE attack (Galvis-Montes et al., 2023). Kininogen is a part of the coagulation cascade (Sparkenbaugh et al., 2020), and the coagulation cascade-related genes are highly upregulated in the post-SE CA1 (Galvis-Montes et al., 2023). Interleukin-6 (IL-6), which is also increased in the CA1 during the early phase of post-SE (Galvis-Montes et al., 2023), is a master regulator of microglia immunomodulation in early epileptogenesis (Morin-Brureau et al., 2018) and is known to regulate the activity of HK (Henderson et al., 2021; Sparkenbaugh et al., 2020). IL-6 also regulates edema formation by influencing astrocytes in the brain following TBI (Xu et al., 2014). Both cytotoxic edema and vasogenic edema are contributor of neuronal hyperexcitability. Cytotoxic edema are serious complications of ischemic and traumatic brain injury (Glykys et al., 2017), and are evident in the early phase of brain injuries (Glykys et al., 2017) and SE (Itoh et al., 2015). Both in the neurons and astrocytes, edema can cause cell swelling due to excess water entry and thus increasing cell volume, which may lead to dysregulated ion homeostasis, sodium and chloride ions, for example, causing neuronal depolarization (Glykys et al., 2017). Damage to the BBB, infiltration of leukocytes and inflammatory mediators are main driving force for cerebral edema (Glykys et al., 2017; Solar et al., 2022). Entry of the plasma proteins into the brain parenchyma may cause vasogenic edema which is partly attributed to the effects of BBB endothelial cells (Takata et al., 2021). Endothelial cells are an integral part of the BBB structural unit, and damage to these cells causes a leaky BBB. Components of the kininogen family including cHK has been shown to induce endothelial cell apoptosis (Zhang et al., 2002). Bradykinin, on the other hand, as an outcome of “kininogen consumption,” has been shown to cause vasogenic edema (Unterberg and Baethmann, 1984), which could be partly attributed to its damaging effects on the (Marcos-Contreras et al., 2016). A possible association of kininogen with brain edema has also been reported in the acute phase of ischemic stroke in a mouse model (Langhauser et al., 2012). In our overexpressed animals, the high levels of kininogen and bradykinin or even cHK formed due to cleavage of kininogen following the epileptogenic stimuli could have caused damage to the endothelial cell layers of the BBB, and thus possibly facilitating quicker entry of the epileptogenic drugs used for modelling in this study, resulting in quickened seizure onset (Figures 4E, 5B). The same also may allow for influx of peripheral inflammatory components into the brain further worsening BBB integrity and ion homeostasis, caused by possible edema formation. All of these may make sense where kininogen, with other possible family members (Aronica et al., 2001), is attributed to the deleterious effects of peripherally-initiated diseases (Sparkenbaugh et al., 2020) or stimuli, thus triggering CA1 hyperexcitability and damage (Wang et al., 2016; Jiang et al., 2023), and thus contributing to epileptogenesis.

### 4.3 Kininogen’s release of bradykinin: a modulator of neuronal firings in both glutamatergic and GABAergic neurons

Next, we tested the effects of bradykinin overexpression on glutamatergic and GABAergic neurons as potential mechanisms of increased seizure susceptibility induced by kininogen. We hypothesized that the effects seen following kininogen overexpression on seizure susceptibility could be due to heightened bradykinin release from elevated kininogen levels in such regions. Bradykinin, at all the doses tested, reduced seizure thresholds in response to PTZ (Figure 5B). At one of these doses, 0.10 μM, bradykinin has been shown to induce glutamate release from astrocytes, while at the dose of 1.00 μM it has been shown to cause elevated calcium levels both in neurons and astrocytes (Parpura et al., 1994), which may explain why bradykinin at both these doses caused highest effects towards rendering seizure susceptibility (Figure 5B). This might be due to bradykinin (Lim et al., 2008) adding strength to the effects of PTZ (Doi et al., 2009) on glutamate uptake via glutamate transporters. We, in our calcium imaging studies, found that bradykinin at 0.10 μM doses increased the frequency of calcium transients in excitatory neurons following subthreshold PTZ treatment having a peak at 11–15 min. Interestingly, calcium transient frequency in the GABAergic PV^+^ neurons was reduced also to the highest at the same time point. Although such effects reduced over time in both neuronal subtypes, they were still mightier in the bradykinin-treated animals compared to the PBS-treated animals at almost all the time points studied. The reduced effects over time could be due to bradykinin at 0.10 μM having the ability to increase glutamate release only for a certain period of time which decreases over time (Parpura et al., 1995; Liu et al., 2009). Excitatory (CA1) pyramidal neurons are known to receive inhibitory inputs in the epileptic brain (Wittner et al., 2005). This may explain why we observed heightened PV inhibitory effects during the first 5-min-window after PTZ treatment in the bradykinin-treated animals (Figure 6C), which could be due to an attempt made by the inhibitory circuits to suppress the heightened excitatory activity (Figure 6B) at the beginning of the pro-epileptic stimuli. Pentylenetetrazole itself is a GABA_A_ receptor antagonist that causes PV^+^ neuronal loss both in the hippocampus and the cortex following seizures (Putra et al., 2020), suggesting its suppressive effects on this subtype of GABAergic neurons. Additionally, a GABA-current-suppressive effect of bradykinin (Lu et al., 1998), concurrently with exclusive effects of PTZ on PV^+^ neurons, may have caused a sharp fall in their inhibitory effects, at least in the 11–15-min time window (Figure 6C). However, GABA activity in the bradykinin-treated animals again seemed to have recovered to the levels of PBS-treated animals in the following time points, possibly due to the usage of a subthreshold dose of PTZ. In the behavioral studies, however, no such recovery was possible due to the addition of further PTZ shots (Figures 4B, 5A), allowing for persisted inhibition and/or death (Ueno et al., 2019) of GABAergic neurons, and thus rendering reduced seizure onset in kininogen- and bradykinin-treated animals (Figures 4E, 5B). To verify this, like in the behavioral study, administration of additional of PTZ shots in the calcium imaging study would be greatly desired, which, however, would be highly challenging with the current imaging setup in our laboratory. Moreover, although our calcium imaging studies were limited to the cortical area, we may assume a similar pattern throughout the brain due to the fact that bradykinin was delivered through the ICV space which is supposed to spread through the whole brain and thus may also exhibit similar effects in the hippocampus (Unterberg and Baethmann, 1984). This, however, should be further verified.

### 4.4 Kininogen and bradykinin: new paradigms in the treatment of epileptogenesis

The downstream molecules of kininogen and bradykinin, including nitric oxide (NO), and B1R and B2R, have also been reported to be associated with epilepsy. However, epilepsy functional studies pertaining to these molecules are still controversial (Adolfo Argañaraz et al., 2004; Rodi et al., 2013; Banach et al., 2011). For example, NO, which is a well-known regulator of bradykinin activities, is known to be associated with seizures/epilepsy. NO is synthesized by three subtypes of NO synthase (NOS): endothelial NOS (eNOS), neuronal NOS (nNOS), and inducible NOS (iNOS). The role of NO in epileptogenesis is still debatable though the majority suggests a possible worsening effect of the same toward epilepsy, while blocking the activity of the NOSs shows mixed outcomes on epileptic treatment (Banach et al., 2011). For example, while nNOS was found to be increased in epileptic patients in some studies (González-Hernández et al., 2000), it was found to be decreased in some (Leite et al., 2002). Similarly, many agents blocking the activity of NOS have been shown to be anticonvulsive, while some suggest anticonvulsive effects of l-arginine, the donor molecule of NO (Banach et al., 2011). Similarly, functional studies related to the effects of B1R/B2R in epilepsy have produced conflicting results among studies. For example, while one study found B2R to be protective against the development of TLE (Adolfo Argañaraz et al., 2004), another found it to be the opposite (Rodi et al., 2013). Therefore, their potential utility in developing therapies targeting epileptogenesis may be uncertain. Our current report thus suggests that kininogen and bradykinin could be better and more precise targets for epileptogenesis.

### 4.5 Limitations and future perspectives

Although we provided herein possible circuitry effects of kininogen through bradykinin’s aggravating activity during seizures, it would be interesting to additionally uncover the related molecular mechanisms by which kininogen might contribute to the course of epileptogenesis. One of the foremost mechanisms that may come into play is inflammation of the brain driven by kininogen. Proinflammatory cytokines, including interleukin-1beta (IL-1β), IL-6, and tumor-necrosis factor-alpha (TNF-α), have been known to increase following seizures. Functionally, IL-1β, for example, decreases GABA-mediated neurotransmission by ∼30% and contributes to neuronal hyperexcitability (Roseti et al., 2015). A decrease in near similar intensity for calcium transients in GABAergic PV^+^ neurons was also observed in this study (Figure 6C). TNF-α, which is increased in the brain after epileptic stimuli, causes aggravation of seizures with worsening electroencephalography profiles (Shandra et al., 2002). Interestingly, both Il-1β and TNF-α have been reported to induce kininogen and bradykinin receptors (Yayama et al., 2000). In TLE patients, IL-6 levels are found to be high (Liimatainen et al., 2009), so are bradykinin levels (Simões et al., 2019). IL-6 participates in the activation of *Kng1* gene (Chen et al., 1991), and participates in kininogen-mediated chronic inflammation and mortality (Sparkenbaugh et al., 2020). In contrast, when kininogen is depleted in mice, the levels of these cytokines are downregulated in an effort to contain inflammatory burden and disease severity (Köhler et al., 2020), suggesting an interplay among these molecules in regulating inflammation and related organ damages. Therefore, it could be a possibility in the context of this study that cytokines such as IL-1β, IL-6, and TNF-α may induce the effects of kininogen and/or *vice versa* following an initial trigger (the first dose PTZ, in our case) to aggravate the subsequent effects and thereby causing increased susceptibility. Another possible reason, yet with a causal relationship with inflammation, could be a damaging effect to the BBB, possibly mediated directly by kininogen and by bradykinin in through downstream pathways. Seizures cause BBB leakage by disrupting the tight junction, and dysfunctional BBB is associated with worsened epilepsy outcomes (Greene et al., 2022). Neurodegeneration due to a combined effect of BBB damage and inflammation can be mediated by kininogen (Langhauser et al., 2012). Bradykinin, through its receptor, B1R, promotes brain microvascular endothelial cell permeability (Huang et al., 2022), and mediates the effects of hyperfibrinolysis on BBB damage (Marcos-Contreras et al., 2016). Therefore, there could be a loop where elements such as epileptic stimuli, inflammation, and BBB damage are mediated in concert with proinflammatory cytokines to induce *Kng1*, possibly by further releasing bradykinin and activating the bradykinin receptor signaling to cause reduced activity of GABAergic neurons and heightened activity of excitatory neurons. It should be noteworthy, however, that although bradykinin mediates most of the pathophysiological effects of kininogen, it may not always be true. For example, the protective effects of HK deficiency against coagulation and inflammation in SCD model mice are not mediated by bradykinin signaling (i.e., B1R/B2R) (Sparkenbaugh et al., 2020). Microglial phagocytosis of amyloid beta, as another example, can be a lot stronger in cells treated with HK or cHK, while bradykinin’s effect is relatively much smaller (Zamolodchikov et al., 2022). These suggest that many pathophysiological effects of kininogen may sometimes be exclusive to itself rather than always be related to its release of bradykinin and the other downstream molecules, and the effects seen in our study individually mediated by kininogen or bradykinin could either be interrelated or not be related at all. Understanding such relations, if any, in the context of epileptogenesis in a better depth would be necessary. Importantly, although one of our previous studies (Zou et al., 2019), along with the current report, and another study from another team of researchers (Simões et al., 2019) have found some association of kininogen levels in human plasma and CSF from epileptic patients and animal models of epilepsy, there is no information on the brain tissue expression patterns of kininogen in post-mortem brains from epilepsy patients–an aspect which should be discretely studied and reported. Moreover, correlation data of such findings along with plasma and/or CSF levels against patient mortality data would be useful, which are absent in the current report. Another major drawback of the current study remains in the inability of performing calcium imaging from the hippocampus. Within the scope of the imaging setup in our laboratory, imaging of cortical layers up to a depth of 0.5 mm from the dura surface was possible, while imaging from the hippocampus may require modern-day miniature imaging systems that allow for imaging from deeper structures beyond the cortical layers. Therefore, employing such techniques in future studies to gather an insight on the hippocampal excitability profile of kininogen- or bradykinin-aggravated seizure susceptibility would be much needed. Moreover, studying the role of kininogen overexpression (and/or knockout) beyond the hippocampal CA1 area in epileptogenesis would be interesting.

## 5 Conclusion

This serves as a foundational report study on the role of neuron-derived kininogen and its subsequent release of bradykinin in the pathogenesis of epileptogenesis. It also offers promising avenues for developing therapies to prevent or mitigate epileptogenesis.

## Data Availability

The scRNA-seq datasets used herein for bioinformatic analyses are publicly available from the original authors at https://zenodo.org/record/8041323, https://zenodo.org/record/8041114, and https://doi.org/10.1101/2022.03.17.484798 (Zeng et al., 2023; Ana, 2022).

## Supplementary material

SUPPLEMENTARY VIDEO S1 Representational seizure (stages) induced by pentylenetetrazole during a seizure susceptibility test, which was preceded by neck jerks (Grade III) for the first 6-8 sec followed by transformation to bilateral forelimb clonus (Grade IV, ∼8-28 sec), followed by generalized tonic-clonic seizure (Grade V, ∼28-71 sec), followed by post-seizure depression (∼72-139 sec) and recovery (∼140 sec onwards).
